# Making decisions about health information on social media: a mouse-tracking study

**DOI:** 10.1186/s41235-022-00414-5

**Published:** 2022-07-22

**Authors:** Mark Lowry, Neha Trivedi, Patrick Boyd, Anne Julian, Melissa Treviño, Yuki Lama, Kathryn Heley, Frank Perna

**Affiliations:** National Cancer Institute, 9609 Medical Center Drive, Rockville, MD USA

**Keywords:** Health literacy, Social media, Cognition, Mouse tracking, Reaction times

## Abstract

Health misinformation is a problem on social media, and more understanding is needed about how users cognitively process it. In this study, participants’ accuracy in determining whether 60 health claims were true (e.g., “Vaccines prevent disease outbreaks”) or false (e.g., “Vaccines cause disease outbreaks”) was assessed. The 60 claims were related to three domains of health risk behavior (i.e., smoking, alcohol and vaccines). Claims were presented as Tweets or as simple text statements. We employed mouse tracking to measure reaction times, whether processing happens in discrete stages, and response uncertainty. We also examined whether health literacy was a moderating variable. The results indicate that information in statements and tweets is evaluated incrementally most of the time, but with overrides happening on some trials. Adequate health literacy scorers were equally certain when responding to tweets and statements, but they were more accurate when responding to tweets. Inadequate scorers were more confident on statements than on tweets but equally accurate on both. These results have important implications for understanding the underlying cognition needed to combat health misinformation online.

**Supplementary Information:**

The online version contains supplementary material available at 10.1186/s41235-022-00414-5.

## Significance statement

Over 70% of the US population has a social media account, and are susceptible to misinformation online, especially those with inadequate health literacy. For example, misinformation about vaccines on Twitter may make users more hesitant to get inoculated. This study examines misinformation related to health risk topics (vaccines, alcohol and tobacco) on social media. We employed mouse tracking to better understand how information presented as a tweet is processed compared to information presented as a simple statement. Public health researchers will benefit from the finding that inadequate health literacy scorers are overconfident when evaluating Tweets. Conversely, cognitive psychologists can use the results to better understand how intuition and deliberate decision-making interact in real time. The combined results may inform researchers across fields with information to combat the spread of false information online.

## Introduction

The spread of health misinformation is a problem online (Gage-Bouchard et al., 2018), and it is especially problematic on social media sites like Twitter and Facebook (e.g., Fletcher et al., 2018; Kouzy et al., 2020). Health misinformation is defined as “a health-related claim of fact that is currently false due to a lack of scientific evidence” (Chou et al., 2018). It is difficult for social media users to identify uncorroborated health claims given the sheer quantity of information on such platforms (Moorhead et al., 2013). Believing misinformation can influence people to make poor health decisions that can harm themselves or those around them. It creates doubt and uncertainty among social media users when evaluating credible health information, subsequently delaying health actions or enacting potentially harmful health behavior. For example, current research regarding the COVID-19 pandemic has demonstrated that widespread misinformation on social media is associated with problematic real-world behaviors, such as non-compliance with wearing a mask and social distancing guidelines (Bridgman et al., 2020).

In this study, we focus on how people make decisions about information related to three health risk factors: smoking, alcohol and vaccines. For example, it is widely known that smoking causes cancer (e.g., Hecht, 1999). Vaccines are important for controlling disease, and vaccine hesitancy is a problem, especially during the Covid-19 pandemic (e.g., Troiano & Nardi, 2021). Alcohol can damage DNA. For example, one of the by-products of digesting alcohol is acetaldehyde, which can bind with DNA causing genetic mutations associated with cancer (see Centers for Disease Control and Prevention, 2019; LoConte et al., 2018).

Health literacy is an important moderating factor for evaluating health information (Diviani et al., 2015). Health literacy is “the degree to which individuals have the capacity to obtain, process and understand health information” (Ratzan & Parker, 2006). Health literacy is situation dependent and it can evolve over time (Edwards et al., 2012). It is likely related to information processing. For example, the Elaboration Likelihood Model (ELM; Petty & Cacioppo, 1986) posits attitude change depends on one’s motivation and ability to engage with a message and is influenced by an individual’s capacity to process information. There are two routes for message persuasion: (1) central route or (2) peripheral route. The central route encompasses one’s high elaboration and cognitive ability to process message quality. The peripheral route operates when one elaborates through low-effort processes and utilizes heuristic cues to understand a message.

Even though cognitive abilities are related to health literacy, little is known about whether health literacy moderates how people process health information (Boyle et al., 2013). It has been hypothesized that those who score low in health literacy will rely on intuition to process information, whereas, those who score high will assess the truthfulness of information by carefully evaluating it in distinct stages (Chiang & Jackson, 2013). In social media settings, peripheral information may distract users from processing the actual message (Bergstrom & Schall, 2014; Trivedi et al., 2021), and might be especially problematic for those with inadequate health literacy.

### What can mouse tracking tell us about underlying cognitive processes?

In this study, we employ mouse tracking to make inferences about how participants respond in real time to health information. Mouse tracking is an empirical approach that can “expose the micro-structure of real-time decisions” (Freeman, 2018). Mouse tracking not only provides chronometric data (e.g., reaction times), it may also inform researchers about response preference over the course of a trial, whether processing happens incrementally or in discrete stages, and overall response certainty (see Freeman, 2018, for a review). Understanding these basic cognitive factors is becoming ever more important to combat health misinformation (see Chou et al., 2020).

Under the mouse tracking paradigm, participants view a stimulus and make a decision about it by clicking on one of two buttons on the top left and right of the screen. At the start of the trial, the cursor is located in the bottom middle of the screen. As they respond, mouse movements are tracked in three dimensions: x-position, y-position and time. Consider the following example. A participant is given a stimulus “Choose.” On the top right and left of the screen are pictures of ice cream and an apple respectively. The participant is instructed to click on the picture that best aligns with their long term health goals. This procedure was used by Stillman, Medvedev and Ferguson (2017) to understand self-control. Based on the distribution of mouse tracking trajectories, they concluded that self-control is a dynamic process. Goals and temptations compete dynamically, rather than in a discrete stages where impulses are abruptly inhibited. We will now briefly discuss the main dependent variables of our study and what they suggest about cognition.

#### Response preference over time

A basic and useful metric is overall response preference. As participants move their mouse on a trial, the x-position of the cursor is considered a “proxy for a participant’s current absolute preference” (see Kies-lich & Henninger, 2017, assuming response options are on the left and right of the screen). Response preference over time is measured as a function of a participants normalized x-position over normalized time over the course of the trial.

In this study, we normalize the x-position where zero is the x-position of the cursor at the start of the trial, and one is the x-position when the participant clicks a response. Similarly, plotting response preference over time will show at what point participants’ preference emerges.

We expect preference to depend on one’s speed of processing and health literacy. One might expect that those with adequate health literacy also process information more quickly than those with inadequate health literacy (Colter & Summers, 2014). However, adequate health literacy might also cause participants to evaluate information more carefully (i.e., careful reading is related to re-reading, see Schotter et al., 2014), which will in turn increase the time it takes them to make a preference.

#### Discrete or incremental processing

Researchers have often tried to determine the steps involved in decision making. For example, there may be stages for perception, evaluation and motor movement (e.g., Tosoni et al., 2008). Or there may be two systems (i.e., “Dual Systems Theory”) that compete with each other (e.g., Evans, 2008; Neys, 2006; Petty and Cacioppo, 1986). The first system is intuitive and relies on perceptual and contextual information. The second system is deliberative and relies on higher cognitive functions (see Kahneman, 2011). Dual stage processing is most likely to occur on difficult tasks when a deliberative stage is needed (see Rotello & Heit, 2000). Research has shown that those who believe fake news may be relying more on an intuitive system than on a deliberative one (Pennycook & Rand, 2019, 2021). Researchers have tried to assess whether these stages/systems are discrete (i.e., the deliberative system processes information after the intuitive system has finished its evaluation) or whether evidence is evaluated incrementally (i.e., the intuitive and deliberative systems overlap as a decision is made).

Plotting the distribution of mouse movements can reveal whether decision making happens incrementally or in discrete stages. It is generally assumed that bimodal distributions of mouse trajectories indicate dual-stage processing (see Freeman, 2018; Freeman & Dale, 2013). For example, Freeman and Dale (2013) argue that measures of bimodality can distinguish between dual and single processing models. Under this view, there are two discrete modes related to an intuitive, quick system and a deliberative, slow one. One mode represents responses where participants head in the right direction from the beginning of the trial (i.e., after initial/intuitive processing, no course correction is needed once the deliberative stage has completed). The other mode represents responses where participants head in the wrong direction at first, but a sudden course correction happens by the deliberative stage.

Conversely, if the distribution is unimodal, then the decision making likely happens incrementally as the statement is being processed. In other words, the deliberative stage happens concurrently with the intuitive stage and the cursor moves gradually to the preferred answer over time.

One common mouse tracking metric to assess bimodality is “Area Under the Curve” or AUC. AUC is the geometric area outlined by the mouse’s real trajectory and the straight line between the mouse’s starting position and response button. Stillman, Medvedev and Ferguson (2017) used AUC to test whether self-control is a dual process where initial temptations need to be suppressed by a more deliberative system. Hartigan’s Dip Statistic (HDS) is often used to determine if the distribution of AUC scores is unimodal or bimodal (e.g., Pfister et al., 2013). HDS tests the null hypothesis that the AUC distribution is unimodal. A rejection of the null hypothesis would indicate bimodality.

We do not expect health literacy to moderate the distribution of AUC. Research into reading has revealed that processing can happen quickly and incrementally (e.g., Rayner & Clifton Jr, 2009), and a few mouse tracking studies have shown that decision making is an incremental process (e.g., Koop, 2013). However, discrete processing cannot be ruled out, especially if trials are complex. For example, Duran (2011) found evidence of dual stage processing when reading negated sentences, and Koop and Johnson (2013) found evidence that a deliberative system can suddenly override an intuitive risk averse system. Overall, we still expect HDS to show unimodal AUC distributions, indicating that the two systems work in parallel.

#### Response confidence

To measure confidence in the response, we use x-position flips. X-position flips are the number of directional changes along the x-axis as participants respond. If a participant’s cursor starts at the bottom and goes straight to the left response button, there would be zero x-flips. If the cursor starts moving toward the right button and then changes direction and clicks the left button, there would be one x-flip.

Koop and Johnson (2013) suggest that x-flips represent instability in the response, where more x-flips indicate greater instability. We take a similar approach, and interpret more x-flips with less certainty (i.e., if a participant is not confident in their answer, they will switch directions more often). This interpretation is supported by Cheng and González-Vallejo (2017), who conducted a principle components analysis of mouse tracking metrics. They found that general decision difficulty comprises two components: conflict and uncertainty. Uncertainty is represented by the number of x-flips in a trial, whereas conflict is represented by average deviation.

A priori, we did not know how health literacy would affect response certainty. On one hand, those with adequate health literacy may have an advantage in overall processing ability, allowing them to make decisions more quickly and confidently. However, there is also evidence that poor performers are overconfident in their abilities (e.g., Kruger & Dunning, 1999). Those who score low in health literacy might also be overconfident even when they respond incorrectly. Additionally, when presented with health information as a tweet, inadequate health literacy scorers may rely on peripheral cues as they respond rather than critically evaluating the information as proposed by ELM (Petty & Cacioppo, 1986). This might inflate their confidence.

### Goals of the current study

The purpose of this study is to deploy a mouse-tracking experiment in Qualtrics [based on code provided by Mathur and Reichling 2019] to assess whether information is processed differently on social media than information presented by itself, and to understand whether those cognitive processes are moderated by health literacy. Mouse tracking can help reveal whether comprehension and decisions about health information are processed in incremental or distinct stages. If incremental processing occurs, a smooth path of the cursor is expected from the stimulus to the chosen response. If the two processes happen in different stages, more irregular/bimodal cursor movements are expected. Additionally, mouse tracking can reveal how response preference changes over time and overall stability of (i.e., confidence in) the response. Thus, we aim to experimentally test:

1. How quickly and accurately participants develop a preference when responding to health information presented as statements and tweets (as measured by response accuracy, normalized x-position over normalized time and overall reaction time),
2. Whether comprehension and decision making about a health message’s credibility happen in two distinct stages or in parallel (as measured by the Hartigan Dip Statistic on area under the curve measurements),
3. How confident participants are in their responses (as measured by x-flips), and
4. Whether health literacy moderates the results of 1–3.

## Methods

### Experiment 1

#### Participants

A convenience sample of 200 US adults (47% female) was recruited through the online participant pool Prolific (prolific.co), and were compensated based on Prolific’s recommendations. Participants were all right handed and had normal or corrected to normal vision. They also had to use a computer for the study (i.e., touch screen devices like phones and tablets were not allowed). Average age was 34.3 years. Funding for participant recruitment was provided by the National Cancer Institute. Sample size was based on simulations in R to provide 0.8 power using an alpha level of 0.05 on the response preference over time analysis.

We assumed that over the entire course of the trial, the average effect size between inadequate and adequate scorers would be small (Cohen’s *d* = 0.2). However, we also accounted for the fact that at the start and end of a trial there would be no difference between scorers. Over the course of the trial, the effect size would grow until Cohen’s *d* reached 0.4. Based on those results, we estimated that 90 participants would be sufficient. However, due to the fact that a disproportionate number of participants on Prolific score adequately in health literacy compared to inadequately, and to account for the fact that those using laptop track pads would be removed from the final sample, we recruited 200 total participants. After removing participants ($n=49$) who used a track pad or whose browser size made it impossible to see the stimulus and response buttons without scrolling ($n=5$), 146 participants were included in the final sample. Approval for the study was given by the NIH IRB.

**Fig. 1:**
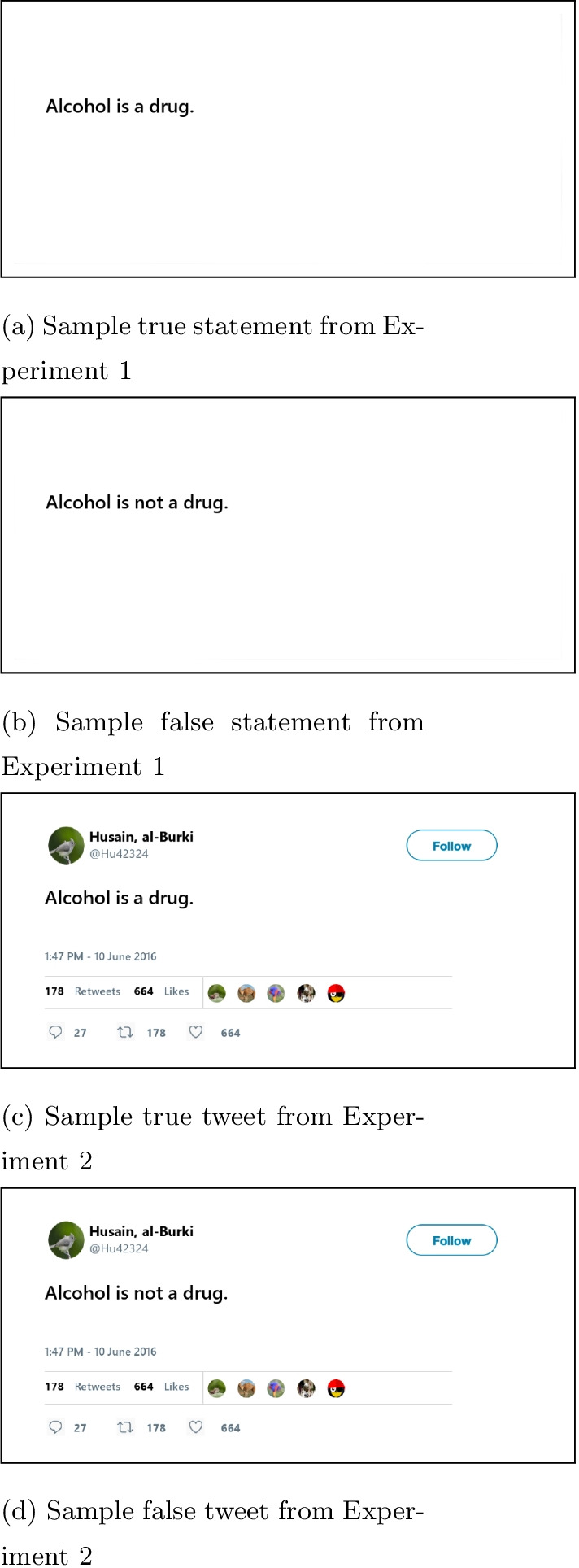
Example stimuli presented in Experiments 1 and 2

#### Materials and measures

##### Health literacy questionnaire

Participants answered the first four questions of the Newest Vital Sign (Weiss et al., 2005) to assess health literacy. The Newest Vital Sign is a quick assessment of health literacy that measures how well a patient can interpret a nutrition label. Participants must answer at least half the questions correctly to obtain an adequate score. There are six questions in total, however whether a participant gets the fifth question correct depends on a verbal answer on the sixth question. In general, if the first four questions are answered correctly, the fifth and sixth questions are not administered. Given that those two questions are best asked in person, and similar to Trivedi et al. (2021), we only administered the first four questions. Those who answered two or fewer questions correctly were classified as “Inadequate Scorers” and those who answered three or four correctly were classified as “Adequate Scorers.”

##### Health statements

We created 60 pairs of health statements (sixty evidence-based; sixty non-evidence-based; see Fig. 1a, b for an example pair). When developing the evidence-based statements, we used information available on the CDC website (e.g., https://www.cdc.gov/alcohol/index.htm), and tried to keep them short (less than 50 characters). After the evidence-based tweet was created, a word or phrase was changed to create a non-evidence-based complement. The health statements referred to one of three topics: vaccines, alcohol or smoking. Text was positioned in the same place that text in a tweet would be (i.e., compare Fig. 1a–c). Participants saw thirty evidence-based and thirty non-evidence-based statements for a total of sixty trials. If a participant saw an evidence-based statement, then they were not shown the corresponding non-evidence complement. The version of each statement (i.e., evidence-based/non-evidence-based) was counterbalanced across participants to control for stimulus complexity between the true and false statements. The statements used can be found in the Appendix.

##### Response preference over time

Recall that response preference over time is measured as a function of a participant’s normalized x-position over normalized time over the course of a trial. In this study, we normalize the x-position where zero is the x-position of the cursor at the start of the trial, and one is the x-position when the participant clicks a response. Similarly, time is normalized into 51 “timesteps” from the start of the mouse movement to the time that participants click a response. This was done in order to average trajectories across several trials. Generally in mouse tracking studies, researchers normalize data into roughly 100 time steps (e.g., Scherbaum & Kieslich, 2018). However, due to the decreased sampling frequency of mouse positions of online studies, we have opted to use 51 instead. Plotting response preference over time shows at what point in a trial participants’ preference emerges.

A Bayesian Hierarchical Model (BHM) run in rjags (Plummer, 2013) was used to analyze how average x-position changed over time for adequate and inadequate health literacy scorers. The x-position was input as the dependent variable. Health literacy (*adequate or inadequate scorers*) and time step (*1–51*) were input as independent variables. The model also controlled for participant and text of the statement. The BHM assumes that the x-position during a given time step comes from a normal distribution, and it provides posterior distribution estimates of each independent variable’s effect on the normalized X-Position. If 95% of the highest density interval (HDI) of the posterior distribution estimate does not include zero, then the effect is considered credible. HDIs are analogous to confidence intervals in traditional null hypothesis testing. Using HDIs, the model can be used to estimate deflections (i.e., how much a condition is different from the grand mean) and mean differences.

##### Overall accuracy

Accuracy was determined based on whether participants made a correct decision about a statement or tweet’s veracity. A correct decision was coded as 1, and an incorrect decision was coded as 0.

Number of trials correct out of sixty for each participant was input into a Bayesian Hierarchical Model (BHM) using rjags. The model assumed a binomial distribution, and estimates how the independent variables affect accuracy in terms of percentage correct. Health literacy (*adequate vs. inadequate scorers*) and experiment (*statements vs. tweets*) were input as independent variables.

##### Response time

The time in milliseconds it took for participants to click a response button from the beginning of each trial was used to calculate response times.

Response times were analyzed using a Bayesian hierarchical model (BHM) using rjags. The BHM assumes that reaction times come from an ex-Gaussian distribution, and it provides posterior distribution estimates of each independent variable’s effects on naming latencies in milliseconds. Health literacy (*adequate or inadequate scorers*) and Experiment (*statements vs. tweets*) were input as independent variables. The model also controlled for participant and text of the statement.

##### Bimodality

Recall that bimodal distributions indicate dual-stage processing while unimodal distributions indicate decision making likely happens incrementally. Area under the curve (AUC) distributions can be used to determine the amount of overlap between cognitive stages (see Freeman & Dale, 2013). AUC measures the “geometric area between the observed trajectory and the direct path” (see Kieslich et al., 2019). If the participant moves the mouse directly to the response button on a trial, AUC for that trial would be zero. Hartigan’s Dip Statistic (HDS) is used to statistically test for bimodality of the response distribution of AUC. If the null hypothesis is rejected in an HDS analysis, then the AUC distribution is considered bimodal.

##### Confidence in the response

To measure confidence in the response, we use x-position flips. Recall that x-position flips are the number of directional changes along the x-axis as participants respond throughout a trial. More x-flips indicate greater uncertainty.

A Bayesian hierarchical model, was used to analyze x-flips on each trial. Health literacy (*adequate or inadequate scorers*) and Experiment (*statements vs tweets*) were input as independent variables. The model also controlled for participant and text of the statement.

#### Procedure

Participants answered demographic information and completed the experiment on Qualtrics (url: Qualtrics.com). The main mouse tracking experiment and code were based on methodology outlined in Mathur and Reichling (2019). During the main experiment, participants were asked to determine as quickly and accurately as possible whether health statements were true or false. On each trial, participants saw the statement outlined in black centered at the bottom of the screen. Buttons labeled *True* and *False* were positioned at the top of the page, either on the right or the left, and the position of the buttons was counterbalanced across participants. Once the statement loaded, participants had 2.5 s to read it before being prompted to move their mouse. The reading time was informed by Brysbaert (2019). Participants then had eight seconds to indicate if the statement was true or false by clicking on the corresponding button. After clicking the response button, participants clicked on the next button to start the next trial. To ensure participants understood the task, they also completed six practice trials, and a GIF was shown that provided an example of how they should respond. Additionally, prompts indicated whether participants moved their mouse before the statement loaded, or whether they took too long to respond. Mouse movements, clicks and whether the participant received a prompt were recorded.

### Experiment 2

#### Participants

A convenience sample of 200 US adults (51% female) was recruited through the online participant pool Prolific (prolific.co), and were compensated based on Prolific’s recommendations. Average age was 34.5 years. Funding for participant recruitment was provided by the National Cancer Institute. Sample size was based on the same power analysis in Experiment 1.

Due to the fact that a disproportionate number of participants on Prolific score adequately in health literacy compared to inadequately, and to account for the fact that those using laptop track pads would be removed from the final sample, we recruited 200 total participants. After removing participants ($n=31$) who used a track pad or whose browser size made it impossible to see the stimulus and response buttons without scrolling ($n=5$), 162 participants were included in the final sample Approval for the study was given by the NIH IRB. Participants were all right handed and had normal or corrected to normal vision.

#### Materials and measures

The materials and measures were the same as in Experiment 1, except the statement pairs were replaced with tweet pairs. The tweets were created in the Magick package (Ooms, 2018) in R. Tweets had the same text as the statements. Pairs of tweets looked identical, except for the text, which determined if if was an “evidence-based” or “non-evidence-based” tweet (see Fig. 1c, d for an example of a tweet pair; see https://osf.io/28xzd/?view_only=eed642fab7624841b9644ef82ecfd65d for the stimuli used).

#### Procedure

The procedure was identical to Experiment 1, except the statement pairs were replaced with the tweet pairs.

**Fig. 2:**
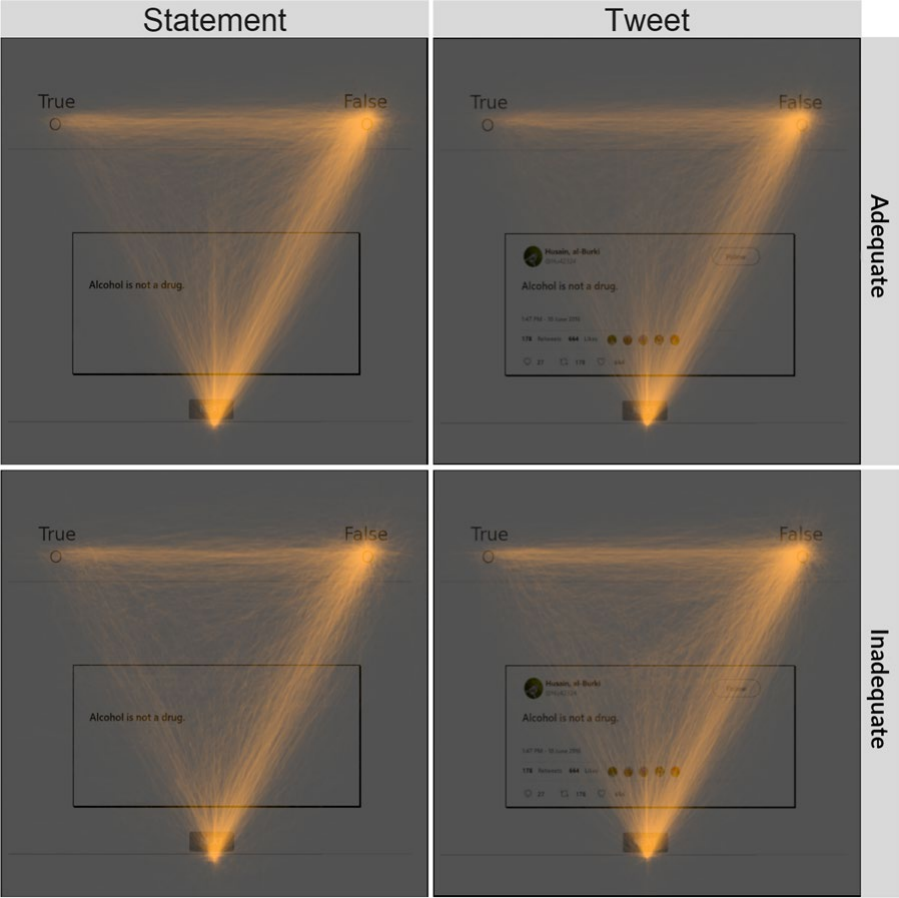
Heat maps of mouse movements by experiment and health literacy. Heat maps are shown for statements used in Experiment 1 (left panels) and tweets in Experiment 2 (right panels). The heat maps show incremental processing for many trials (i.e., there are smooth paths from the start button to the response button). However it is also apparent that overrides exist in the data (i.e., abrupt changes occur, especially near the response buttons). These overrides indicate that dual stage processing may happen late in the trials

**Table 1:** Descriptive statistics—uncertainty and bimodality for Experiments 1 and 2

	X-position flips					Hartigan dip statistic for AUC			
	Statement adequate	Statement indadequate	Tweet adequate	Tweet inadequate		Statement adequate	Statement indadequate	Tweet adequate	Tweet inadequate
*N*	97	47	102	62		97	47	102	62
Mean	2.1	2.8	2.3	2.3	HDS	0.002	0.003	0.002	0.003
SE	0.03	0.06	0.03	0.04	*p* value	0.99	0.99	0.99	0.99
Median	2	2	2	2

**Table 2:** Descriptive statistics—reaction time measurements for Experiments 1 and 2

	Reaction time				Initiation time			
	Statement adequate	Statement indadequate	Tweet adequate	Tweet inadequate	Statement adequate	Statement indadequate	Tweet adequate	Tweet inadequate
*N*	97	47	102	62	97	47	102	62
Mean	2689	3135	2808	3230	926	1073	992	1202
SE	13	23	13	21	9	17	9	16
Median	2490	2933	2603	2985	880	1015	973	1097

## Results

Mouse trajectories were screened and prepared in R (R Core Team, 2013) using the MouseTrap package (Kieslich & Henninger, 2017). Of the 18,600 trials from participants in both experiments, 288 (1.5%) were excluded based on irregular responses (e.g., taking too long to respond, not moving the mouse soon enough, etc.).

### Descriptive statistics

#### Experiment 1

Overall, adequate health literacy scorers answered 84% of the trials correctly, whereas inadequate health literacy scorers answered 80% of trials correctly.^1^ Descriptive statistics for mouse movements (e.g., Area Under the Curve and number of X-Position Flips) are given in Table 1. Descriptive statistics for total response time and initiation time are given in Table 2.

#### Experiment 2

Overall, adequate health scorers answered 88% of the trials correctly, whereas inadequate health literacy scorers answered 79% of trials correctly. Descriptive statistics for Area Under the Curve and number of X-position flips are given in Table 1. Descriptive statistics for total response time and initiation time are given in Table 2.

**Fig. 3:**
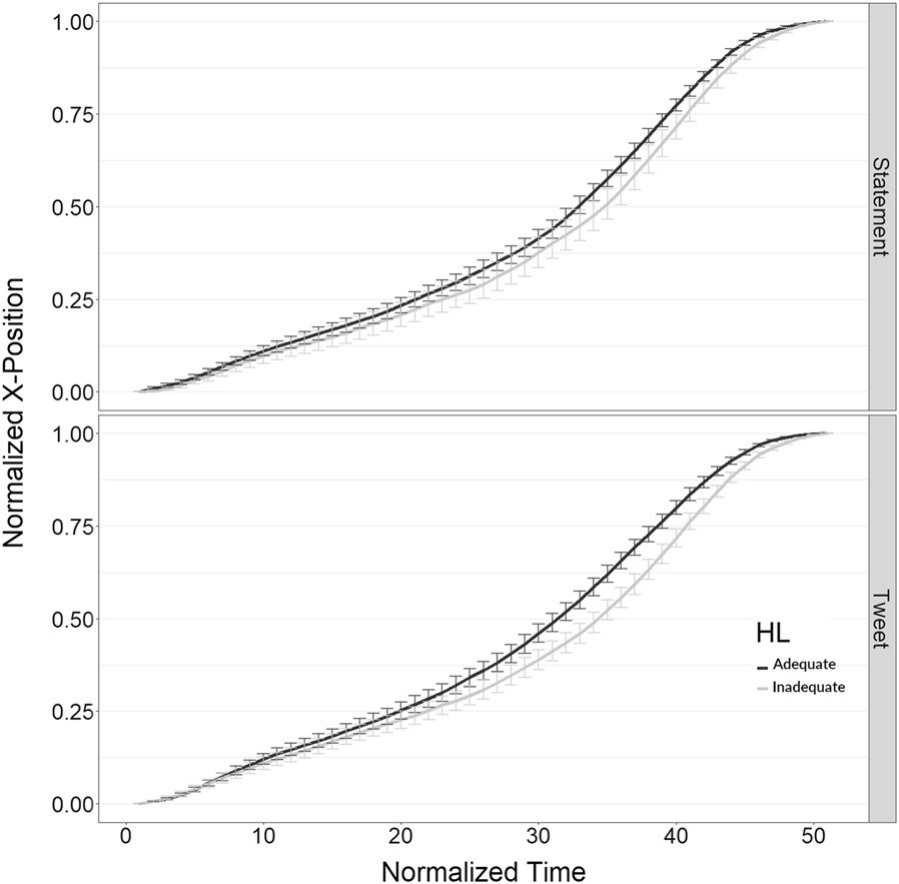
Normalized X-position by time step. The top panel shows results for statements (Experiment 1). The bottom panel shows results for tweets (Experiment 2). Adequate health literacy scorers are represented by the black line, while inadequate health literacy scorers are represented by the gray line. Adequate scorers made their preference earlier in the trial compared to inadequate scorers for both tweets. Error bars show standard errors

### Position by time step analysis (response preference over time)

#### Experiment 1

There was no main effect of health literacy: adequate scorers were on average only 0.03 units (out of 1) closer to the response button than inadequate scorers. There was no interaction between health literacy and time step. No analysis was used to assess the main effect of time steps because each trial started at 0 and ended in 1. See Fig. 3 (top panel) for a graph of the results and Additional file 1 for the BHM of the results.

#### Experiment 2

Overall, there was a main effect of health literacy: adequate scorers’ cursors were 0.04 units (out of 1) closer to the response button compared to inadequate scorers on any given time step, 95% HDI [0.01, 0.08)]. There was also an interaction between health literacy and time step: adequate scorers were credibly different from inadequate scorers on time steps 26–43. See Fig. 3 for a graph of the results and Additional file 2 for a BHM of the timestep results.

**Fig. 4:**
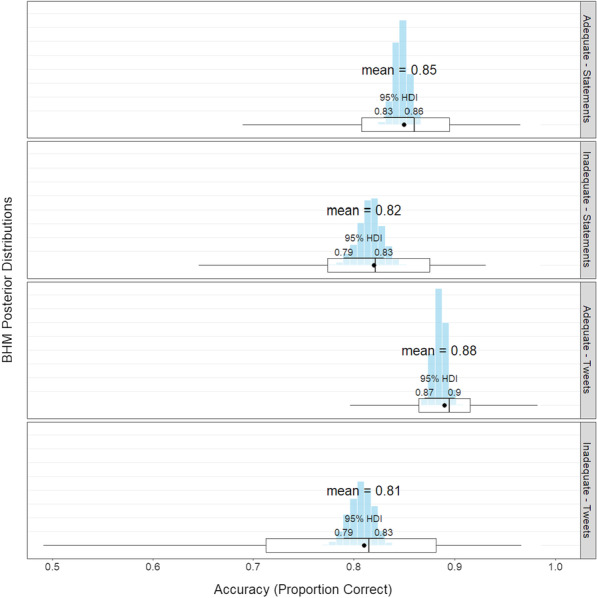
Accuracy by health literacy and experiment. The blue histograms represents the mean’s posterior distribution. On top is the most likely mean value based on the BHM. In the middle is the 95% highest density interval. At the bottom is a box plot based on the raw data averaged by participant (the black dot represents the raw mean)

### Overall accuracy

Overall, there was a main effect of health literacy: adequate scorers were 5.4% more accurate than inadequate scorers, 95 % HDI [3.4, 7.2]. To ensure that NVS was a sensitive measure of health literacy, we also calculated the Bayes Factor for the main effect using the Savage–Dickey density ratio (Lee & Wagenmakers, 2014). $\hbox {BF}_{10}= 1864$, indicating very strong support in favor of the alternative hypothesis (i.e., accuracy scores are different for adequate and inadequate scorers). There was no main effect of experiment (95% HDI [$-6.8, 0.6$]).

There was a credible interaction: adequate scorers were 3.8 % *more* accurate on tweets than on statements, 95 % HDI [2.1, 5.7]; however, inadequate scorers were 0.9% *less* accurate on tweets than statements 95% HDI [$-2.2, 4.1$]. See Fig. 4 for a graph of the interaction.

To better understand how inadequate and adequate scorers performed relative to each other in tweets and statements, we performed additional comparisons. For statements, adequate scorers were 3.1% more accurate than inadequate scorers, 95% HDI [0.6, 5.8]. For tweets, adequate scorers were 7.7% more accurate than inadequate scorers, 95% HDI[5.1, 10.1].

#### Exploratory analysis: d-prime and decision criteria

Given that this is a two alternative forced choice design, it is possible that participants were more discriminating on tweets than statements, or that they have a response bias toward true statements. We measured d-prime (*d’*) and the criterion ($\beta$) for each participant and then conducted a Bayesian ANOVA in JASP (JASP Team, 2021) with health literacy and experiment as independent variables.

For d-prime, there was a main effect of health literacy ($\hbox {BF}_{10} =14.06$): adequate scorers ($d'=1.20$, 95% HDI [1.10, 1.28]) were better able to discriminate between true and false tweets than inadequate scorers ($d'=0.95$, 95% HDI [0.83, 1.08]). There was no main effect of experiment or interaction between health literacy and experiment.

For the criterion, we also found a large main effect of health literacy ($\hbox {BF}_{10}= 31{,}957.7$): adequate scorers ($\beta =0.60$, 95% HDI [0.57, 0.63]) were more likely to click the “True” button than inadequate scorers were ($\beta =0.72$, 95% HDI [0.69, 0.76])^2^. There was no main effect of experiment or interaction between health literacy and experiment.

**Fig. 5:**
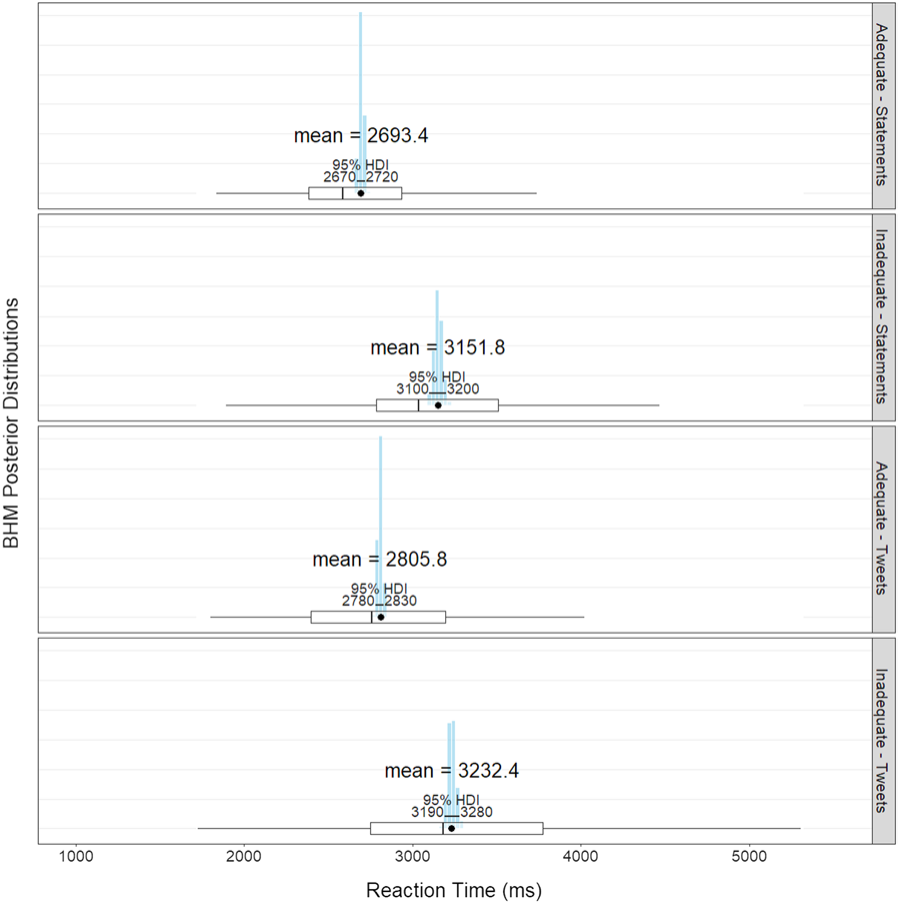
Reaction time by health literacy and experiment. The blue histograms represents the mean’s posterior distribution. On top is the most likely mean value based on the BHM. In the middle is the 95% highest density interval. At the bottom is a box plot based on the raw data averaged by participant (the black dot represents the raw mean)

### Response time

Overall, there was a very large main effect of health literacy: adequate scorers were 442 ms faster than inadequate scorers, 95% HDI [407, 479]. There was also a main effect of Experiment: participants responded to statements 96.5 ms faster than they did to tweets, 95 % HDI [61, 134]. There was no interaction between health literacy and experiment (see Fig. 5).

### Bimodality measures

To test for bimodality in AUC measurements, we computed the Hartigan Dip Statistic (HDS) in R using the MouseTrap package for each Experiment, stratified by Health Literacy. A rejection of the null hypothesis would indicate the distribution is bimodal, suggesting that processing happens in distinct stages. For adequate scorers who viewed statements, $\hbox {HDS} = 0.002$, $p =0.99$. For inadequate scorers who viewed statements, $\hbox {HDS} = 0.003$, $p =0.99$. For adequate scorers who viewed tweets, $\hbox {HDS} = 0.002$, $p =0.99$. For inadequate scorers who viewed tweets, $\hbox {HDS} = 0.003$, $p =0.99$. Overall, HDS measurements of AUC suggest processing of statements and tweets is incremental for both adequate and inadequate scorers. Similar results were found for the most difficult trials (i.e., the 25% slowest and least accurate statements/tweets).

**Fig. 6:**
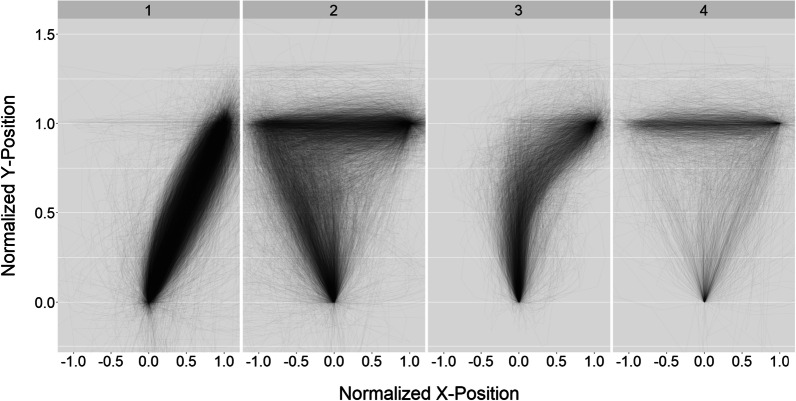
Mouse trajectory clusters. Mouse trajectories were clustered into four groups: (1) Direct Trials where participants make little deviation from the direct path, (2) Late Switches where participants switch direction at the end of the trial, (3) Incremental Trials where processing happens incrementally, and (4) Switchbacks where participants switch directions twice after initially heading in the correct direction

#### Exploratory analyses: clustering mouse trajectories

Upon further inspection of Fig. 2, it is possible that a deliberative stage is overriding a more intuitive stage late in the trials. The participant starts moving in the correct direction, once they get to the response button, they abruptly switch directions and head toward the other response button. Because these overrides are so late in a trial, the area under the curve measurements on overrides are not much different than on trials with no overrides. To further explore this, we categorized mouse trajectories based on their shape using the mousetrap package in R (Kieslich & Henninger, 2017). First, we tested for the optimal number of groupings after spatially normalizing the trajectories. We used the cluster stability method as described in Haslbeck and Wulff (2020) and Wulff et al. (2019). We determined that four groups would be optimal. We then clustered the trajectories using the hierarchical clustering procedure (Ward algorithm) as described in the mousetrap package with default parameters (see Kieslich & Henninger, 2017). Group 1 trials are characterized by relatively little deviation from the direct path (“Direct Trials”; 59% of all participants’ trials on average). During Group 2 trials, participants switch preference abruptly at the end of the trial (“Late Switches”; 24%). Group 3 trials show incremental processing (“Incremental Trials”; 14%). Group 4 trials head in the correct direction initially, switch preference late in the trial, but then switch back (“Switchbacks; 3%). See Fig. 6 for a graph of the trajectory types.

Using this information, we tested the idea, based on ELM, that adequate scorers would rely more on a deliberative stage than an intuitive one compared to inadequate scorers. We expect that adequate scorers would show more *Late Switches* and *Switchbacks* than inadequate scorers since these trials suggest a deliberate stage overriding an intuitive one. For each participant, we calculated the proportion of total overrides by adding the number of *Switches* and *Switchbacks* and dividing by the number of trials. We used a BHM with health literacy score (adequate vs. inadequate) and experiment (tweets vs. statements) as the independent variables.

Contrary to the predictions of ELM, inadequate scorers were *more likely* to have overrides (30.3% of trials) than adequate scorers (26.7% of trials), a 3.6 percentage point difference 95% HDI [0.8, 6.5].

**Fig. 7:**
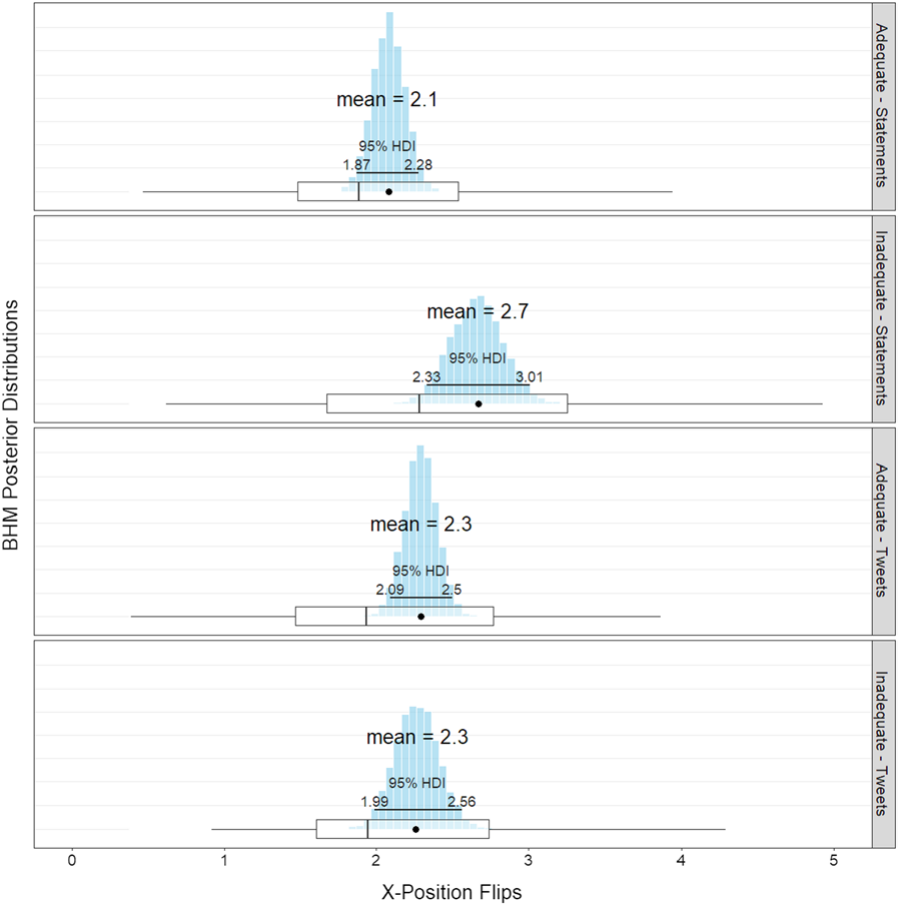
X-position flips—posterior distribution estimates. The blue histograms represents the mean’s posterior distribution. On top is the most likely mean value based on the BHM. In the middle is the 95% highest density interval. At the bottom is a box plot based on the raw data averaged by participant (the black dot represents the raw mean)

### X-position flips

There was a no main effect of health literacy 95% HDI $-0.02$, .58. There was no main effect of Experiment 95% HDI [$-.20$, .40]. However, there was a credible interaction: for statements, inadequate scorers had 0.58 more x-flips on average than did adequate scorers, 95% HDI [0.13, 1.01]; for tweets, inadequate scorers were not credibly different than adequate scorers, 95% HDI [$-0.37$, .42]. This suggests that inadequate scorers become more confident when responding to tweets, but adequate scorers confidence remains the same. See Fig. 7 for a graph of the interaction.

## General discussion

The purpose of this study was to assess how users make decisions about health-related Twitter posts and health-related statements, and whether health literacy was a moderating variable. In general, there is evidence of incremental processing of both tweets and statements. Experiment 1 results indicated that when viewing health statements, both adequate and inadequate scorers develop a preference similarly. Adequate scorers were also more accurate compared to inadequate health literacy scorers in determining the truthfulness of health statements. They were better able to distinguish between true and false information, and had faster reaction times. Thus, the results suggest that adequate health literacy is related to better comprehension and processing of information (see also Meppelink et al., 2015).

In Experiment 2, statements were replaced with tweets, which included contextual information (e.g., profile picture, number of likes, etc.). Adequate health literacy scorers were still more accurate than inadequate health literacy scorers. However, on tweets, adequate scorers start developing a preference earlier during a trial.

We also compared the results of Experiment 1 to Experiment 2. Adequate health literacy scorers were more accurate in assessing the veracity of tweets than statements. Inadequate health literacy scorers were less accurate on tweets than statements, but this difference was not credible (i.e., “significant”). However, based on mouse tracking metrics, how confident participants were in their answers depended on whether they viewed a statement or tweet. Adequate scorers were equally confident when responding to statements and tweets, whereas inadequate scorers were more confident responding to tweets than to statements. In an exploratory analysis, we found that inadequate scorers were more likely to show overrides than adequate scorers. If overrides indicate a deliberative stage reversing the decision of an intuitive stage, then these results are inconsistent with some dual stage models like ELM. ELM posits that inadequate scorers should rely more on a “peripheral route” (i.e., based on intuition/heuristics) than a central route (i.e., based on higher cognitive functions) to process information. They would be less likely to show overrides compared to adequate scorers. Our results likely indicate that inadequate scorers had more difficulty making decisions about the health statements than adequate scorers, possibly due to unfamiliarity or lack of knowledge with the information. However further research is needed to understand why this is the case.

### Speed of processing and response preference

Processing health information as part of a tweet takes longer compared to presenting just the text alone. Also, adequate health literacy scorers answered more quickly than inadequate health literacy scorers in both experiments based on raw reaction times. These results suggest that adequate health literacy scorers processed information more quickly than inadequate health literacy scorers did.

The time step analyses provided additional information. For statements, adequate scorers developed their preference similarly on each trial compared to inadequate scorers. However, on tweets, adequate scorers started developing their preference earlier on in the trial compared to inadequate scorers. One possibility for this is that the contextual information of a tweet distracted inadequate scorers. This kept them from making a preference until later on in the trial. Another possibility is that inadequate scorers were more likely to search contextual information for clues as to how to respond, which delayed their response. These possibilities needs to be explored in future research.

One limitation of our design is that we implemented a static start procedure (i.e., the stimulus appears before participants start moving their mouse). Research has shown that a static start procedure can attenuate effects of continuous measures [e.g., response preference over time, see Scherbaum and Kieslich (2018)]. A dynamic starting procedure (i.e., the stimulus is shown only after a participant moves their mouse) may provide more robust results. However, because our study was conducted online using Qualtrics, we could not control whether the stimulus loads before or after the mouse is moved. Thus, more research may be necessary to confirm our results.

### Incremental processing of health information

The distribution of Area Under the Curve was unimodal for tweets and statements, and it was unimodal for adequate and inadequate health literacy scorers. This suggests that processing the health statements’ meaning and deciding whether it is true happened incrementally, not in distinct stages. This is consistent with research into reading (e.g., Rayner & Clifton Jr, 2009) and is consistent with some mouse tracking studies (e.g., Koop, 2013).

However, the exploratory analysis that clustered mouse trajectories into categories revealed that overrides happen on approximately 25% of trials. These results are consistent with so-called Dual-Stage models where a deliberative stage can override an intuitive stage (e.g., Evans, 2008; Neys, 2006; Petty and Cacioppo, 1986). Thus it seems that although incremental processing may be the norm, dual stage processing does occur. We also found that inadequate scorers were somewhat more likely to have overrides than adequate scorers, which is indicative of dual stage processing and counter to expectancy from ELM. Given that evidence that dual processing is more likely to happen on more difficult trials where a deliberative stage is needed (e.g., Rotello & Heit, 2000), these results likely indicate that inadequate scorers had more difficulty responding.

### Response confidence

One key takeaway from the two experiments is that response confidence is not a good indicator of response accuracy. Based on x-flips, adequate scorers were no more confident on tweets than inadequate scorers, yet they were still more accurate than inadequate scorers. These results suggest that confidence is not a good measure of accuracy.

### Accuracy, response bias and discrimination

Adequate scorers were more accurate overall than inadequate scorers. Additionally, adequate scorers were more accurate on tweets than on statements, but the same was not true for inadequate scorers. The interaction is interesting, and may indicate that adequate scorers are better equipped to evaluate information on social media than inadequate scorers.

Based on exploratory analyses of d-prime, we found that adequate scorers were much better at discriminating between *True* and *False* information than inadequate scorers. Surprisingly, based on the criterion, we found that inadequate scorers were less likely to respond “TRUE” than adequate scorers. This is an unexpected finding, and more research is needed to understand the phenomenon. It is possible that inadequate scorers have greater distrust in messaging from public health institutions. Because we created the stimuli based on statements by the CDC, inadequate scorers’ responses may reflect that skepticism. This is consistent with findings that not trusting the health care system is associated with poor health decisions (Armstrong et al., 2006)

### Communication strategies and health literacy

The spread of health misinformation online is a problem (Chou et al., 2020), and inadequate health literacy scorers may be at risk when evaluating information on social media sites. However, it should be noted that health literacy measures (e.g., the NVS) are not perfect. Health literacy may be the result of other factors, including reading ability, working memory capacity, etc. A user’s health literacy can also evolve over time (e.g., Edwards et al., 2012). Thus, the study is limited in how it generalizes to those with adequate and inadequate health literacy. It may be beneficial for social media companies to remind their users to carefully read a health message. Pennycook et al. (2020) found that simply reminding users to be accurate helped them better discern between true and false information. We expect that such warnings would be beneficial to anyone, especially those with lower health literacy. However, based on these results, simply having users engage a deliberative stage might not be enough (i.e., inadequate scorers were more likely to show dual stage processing, and they were less accurate compared to adequate scorers). Clear, easy to understand health messaging that engenders trust is also important. More research is needed to better understand which communication strategies work, and to disentangle health literacy from other constructs (e.g., working memory).

## Conclusion

Health misinformation on social media is a problem. As it spreads online, people may be susceptible to believing it, leading to poor health choices. In the two experiments in this study, we found that health literacy was a moderating factor in accepting misinformation and in response confidence. Adequate scorers developed a preference more quickly than inadequate scorers. They were also more confident when responding to simple statements. This was not true of inadequate health literacy scorers, despite being more likely to show dual stage processing. It suggests new strategies are needed to combat health misinformation online that takes into account health literacy.

### Supplementary Information

**Additional file 1.** Bayesian Hierarchical Model Results for the Timestep Analysis of Experiment 1.

**Additional file 2.** Bayesian Hierarchical Model Results for the Timestep Analysis of Experiment 2.

## Data Availability

The datasets and materials generated and analyzed during the current study are available in OSF at https://bit.ly/3mMeV2I.

## Appendix

See Table 3.

**Table 3:** Text of statements and tweets used in Experiments 1 and 2

True tweet	Characters true	False tweet	Characters False	Topic
Alcohol is a drug	18	Alcohol is not a drug	22	Alcohol
Alcohol is a depressant	24	Alcohol is a stimulant	23	Alcohol
Alcohol hurts your heart	24	Alcohol use heals your heart	28	Alcohol
Drinking causes impotence	26	Drinking prevents impotence	27	Alcohol
Darker drinks are unhealthy.	28	Darker drinks are healthy	26	Alcohol
Alcohol can damage your DNA	27	Alcohol can’t damage your DNA	29	Alcohol
Drinking alone is not fine	26	Drinking alcohol alone is fine	30	Alcohol
Coffee does not cure a hangover	31	Coffee does cure a hangover	27	Alcohol
You can’t throw up to sober up.	31	You can throw up to sober up	30	Alcohol
Alcohol is processed in the liver	33	Alcohol is processed in the kidney	35	Alcohol
Alcohol use contributes to suicide	34	Alcohol use is unrelated to suicide	35	Alcohol
Alcohol is nonessential to your diet	37	Alcohol is essential to your diet	33	Alcohol
Alcohol withdrawal can be dangerous	35	Alcohol withdrawal can be innocuous	36	Alcohol
A cold shower does not cure a hangover	38	A cold shower does cure a hangover	34	Alcohol
When I seem drunk, I’m not in control	37	When I seem drunk, I’m still in control	39	Alcohol
If you don’t drink, you shouldn’t start.	40	If you don’t drink, you should start.	37	Alcohol
Alcohol is part of an unhealthy lifestyle	41	Alcohol is part of a healthy lifestyle	38	Alcohol
Everybody reacts differently to alcohol.	40	Everybody reacts the same way to alcohol	40	Alcohol
Alcohol slows down activity in the brain	41	Alcohol speeds up activity in the brain	39	Alcohol
Drinking alcohol while pregnant is risky	41	Drinking alcohol while pregnant is safe	39	Alcohol
Vaping is dangerous.	20	Vaping is safe.	15	Smoking
Smoking does kill!	18	Smoking doesn’t kill!	21	Smoking
Vaping is addictive	19	Vaping is non-addictive	23	Smoking
Nicotine is addictive.	22	Nicotine is not addictive	26	Smoking
Nicotine stimulates the adrenal glands	22	Nicotine stimulates hunger hormones	26	Smoking
Second hand smoke is dangerous	30	Second hand smoke is safe	25	Smoking
Cigarettes are still unsafe	27	Cigarettes are much safer now	29	Smoking
Low-tar cigarettes are dangerous	33	Low-tar cigarettes are safe	28	Smoking
Smoking is bad for my lungs	30	Smoking is good for my lungs	31	Smoking
Filters don’t make cigarettes safe	35	Filters make cigarettes safe	29	Smoking
Occasional smoking will hurt me.	32	Occasional smoking won’t hurt me	33	Smoking
Secondhand smoke harms children	31	Secondhand smoke benefits children	34	Smoking
Vaping does not help you quit smoking	37	Vaping helps you quit smoking	29	Smoking
Smoking harms the immune system	32	Smoking benefits the immune system	34	Smoking
Most smokers want to quit smoking	33	Most smokers want to keep smoking	33	Smoking
Nicotine is bad for kids’ brains	33	Nicotine is good for kids’ brains	33	Smoking
Hookah smoke can cause cancer	33	Hookah smoke can’t cause cancer	34	Smoking
There is no safe tobacco product	32	There are some safe tobacco products	36	Smoking
Vaping has been marketed to teens	34	Vaping has not been marketed to teens	37	Smoking
E-cigarettes are not a healthy choice.	38	E-cigarettes are a healthy choice	34	Smoking
Vaccines are safe	18	Vaccines are dangerous	23	Vaccines
Vaccines help everyone	22	Vaccines hurt everyone	22	Vaccines
Vaccines do not cause Autism	28	Vaccines cause Autism	21	Vaccines
Vaccines help prevent illness	29	Vaccines cause illness	22	Vaccines
Vaccines protect communities	29	Vaccines harm communities	26	Vaccines
Vaccinations keep children safe	31	Vaccinations harm children	26	Vaccines
There are no toxins in vaccines	31	There are toxins in vaccines	28	Vaccines
Vaccines are based on science	29	Vaccines are based on ignorance	31	Vaccines
Vaccines help future generations	32	Vaccines harm future generations	32	Vaccines
Vaccines save children’s lives	30	Vaccines don’t save children’s lives	36	Vaccines
Vaccines prevent disease outbreaks	34	Vaccines cause disease outbreaks	32	Vaccines
Vaccines protect future generations	35	Vaccines harm future generations	32	Vaccines
HPV vaccine does not cause infertility	38	HPV vaccine causes infertility	30	Vaccines
Some vaccines are safe for everyone	35	All vaccines are safe for everyone	34	Vaccines
HPV vaccine prevents cervical cancer	36	HPV vaccine causes cervical cancer	34	Vaccines
Vaccines strengthen the immune system	37	Vaccines weaken the immune system	33	Vaccines
Vaccines save people	35	Vaccines Kill People	35	Vaccines
Vaccines are regulated by the FDA	33	Vaccines are not regulated by the FDA	37	Vaccines
The flu shot doesn’t cause the flu	34	The flu shot causes the flu for some	36	Vaccines
Vaccines protect the most vulnerable	37	Vaccines harm the most vulnerable	33	Vaccines

## Footnotes

1 Note: Adequate scorers were more accurate across all three topic areas (Vaccines, Alcohol and Smoking). There was no interaction between topic and health literacy.

2 Note: that $\beta < 1$ for each group, indicating that both groups were biased toward “True”.
